# Amiloride-enhanced gene transfection of octa-arginine functionalized calcium phosphate nanoparticles

**DOI:** 10.1371/journal.pone.0188347

**Published:** 2017-11-16

**Authors:** Juan Ramón Vanegas Sáenz, Taichi Tenkumo, Yuya Kamano, Hiroshi Egusa, Keiichi Sasaki

**Affiliations:** 1 Division of Advanced Prosthetic Dentistry, Tohoku University Graduate School of Dentistry, Sendai, Miyagi, Japan; 2 Laboratory for Redox Regulation, Tohoku University Graduate School of Dentistry, Sendai, Miyagi, Japan; 3 Division of Molecular and Regenerative Prosthodontics, Tohoku University Graduate School of Dentistry, Sendai, Miyagi, Japan; Universidad de Castilla-La Mancha, SPAIN

## Abstract

Nanoparticles represent promising gene delivery systems in biomedicine to facilitate prolonged gene expression with low toxicity compared to viral vectors. Specifically, nanoparticles of calcium phosphate (nCaP), the main inorganic component of human bone, exhibit high biocompatibility and good biodegradability and have been reported to have high affinity for protein or DNA, having thus been used as gene transfer vectors. On the other hand, Octa-arginine (R8), which has a high permeability to cell membrane, has been reported to improve intracellular delivery systems. Here, we present an optimized method for nCaP-mediated gene delivery using an octa-arginine (R8)-functionalized nCaP vector containing a marker or functional gene construct. nCaP particle size was between 220–580 nm in diameter and all R8-functionalized nCaPs carried a positive charge. R8 concentration significantly improved nCaP transfection efficiency with high cell compatibility in human mesenchymal stem cells (hMSC) and human osteoblasts (hOB) in particular, suggesting nCaPs as a good option for non-viral vector gene delivery. Furthermore, pre-treatment with different endocytosis inhibitors identified that the endocytic pathway differed among cell lines and functionalized nanoparticles, with amiloride increasing transfection efficiency of R8-functionalized nCaPs in hMSC and hOB.

## Introduction

Nanotechnology plays a key role in developing the innovative biomaterials required for new therapeutic techniques [1,2]. In biomedicine, gene delivery systems are classified as viral and non-viral vectors. Viral vectors exhibit high transfection efficiency but are associated with immunogenicity, inflammatory response, recombination, and carcinogenicity. Non-viral transfection techniques are considered as powerful alternative gene delivery systems and have emerged from the need to achieve high efficiency and/or prolonged gene expression but with a low toxicity compared to that of viral vectors [3–5]. In particular, owing to their favourable properties such as high biocompatibility and good biodegradability, nanoparticles comprised of calcium phosphate (nCaP), the main inorganic component of human bone and teeth [6–9], have garnered considerable interest as biomedical tools and have been used as bone substitution materials as well as drug carriers and transfection agents.

The ability of nCaPs to serve as transfection agents as an alternative option to viral vectors has been well studied with good results [6–8,10]. The transfection efficiency of nCaP is considerably higher with the incorporation of DNA into a multi-shell structured nanoparticle to overcome several physical and chemical barriers, e.g. intracellular degradation in lysosomes. Furthermore, the addition of targeting moieties such as antibodies or peptides on the outer layer of the nCaP could both protect and release the genetic materials to achieve the therapeutic goals as well as serving to increase the transfection efficiency [11–13]. The properties of nCaPs have thus benefited from the use of different coating agents including polyethylenimine (PEI) [14], protamine [15], and poly(lactic-co-glycolic acid) [16] to improve in some extent the transfection efficiency in particular. However, several issues remain to be overcome including the cell cytotoxicity and cell specificity related to these coating agents.

In comparison, octa-arginine (R8) is a small cationic synthetic peptide that mimics the trans-activator of transcription of the human immunodeficiency virus [17] and has the ability to cross the plasma membrane of eukaryotic cells [18]. R8 has been effectively used as a drug delivery or gene transfer carrier for various nanoparticles and biomolecules owing to its low cellular toxicity [2,19,20]. As such use of arginine-rich peptides appears to improve intracellular delivery systems, the introduction of R8 into nCaP might therefore be expected to also increase the gene transfection efficiency.

To verify this effect, multi-shell nCaPs with different concentrations of R8 were fabricated and their transfection efficiency and endocytic pathway in different cell lines were evaluated. Here, the results of incorporating R8 onto nCaP, which showed different transfection efficiencies among cell lines with particularly high efficiencies in human mesenchymal stem cells (hMSCs), are presented. Cell viability was high in all cells regardless of phenotype, suggesting that R8-functionalized DNA-loaded nCaP might be a good option for gene delivery as a non-viral vector. Furthermore, the endocytic pathway utilized differed among cell lines and concentrations of R8-functionalized nCaP. In particular, R8-functionalized nCaPs showed notably high transfection efficiencies in hMSCs and human osteoblasts (hOBs) that had been treated with amiloride as an inhibitor of endocytosis.

## Materials and methods

### Preparation of nanoparticles

To prepare multi-shell R8-functionalized DNA-loaded nCaP (CaP/DNA/CaP/R8), a previously described procedure was followed [6,14,15]. An aqueous solution of calcium nitrate (Ca(NO_3_)_2_) (18 mM) (Wako P.C.I. Ltd.) was mixed with an aqueous solution of diammonium hydrogen phosphate ((NH_4_)_2_HPO_4_) (10.8 mM) (Wako). The pH of both solutions was adjusted beforehand to 9 with sodium hydroxide (0.1 M) (Wako). Both solutions were mixed into a small beaker using a peristaltic pump in a Ca:P ratio of 1.6:1. From this dispersion, 50 μl was collected with a pipette and rapidly mixed with 20 μl of an aqueous solution of the AcGFP1 plasmid encoding a green fluorescent protein (1 mg ml^−1^) (GenScript, USA Inc.) or a pUC57 plasmid encoding human bone morphogenetic protein (BMP)-2 (1 mg ml^−1^) (GenScript USA), in a sterile Eppendorf tube. This was followed by the addition of 25 μl each of aqueous solutions of Ca(NO_3_)_2_ and (NH_4_)_2_HPO_4_. The outer layer was finally stablished by the addition of 20 μl of different concentrated solutions of R8 (0.1, 1, 5, 10, 50, 100 mg ml^−1^) (Sigma Genosys) to create six different dispersions of CaP/DNA/CaP/R8 nanoparticles.

CaP/DNA/CaP/PEI nanoparticles (control group) were prepared by mixing 50 μl of an aqueous nCaP dispersion (prepared as above) with 20 μl of an aqueous solution of AcGFP1 or pUC57 plasmid in a sterile Eppendorf tube, followed by the addition of 25 μl each of aqueous solutions of Ca(NO3)2 and (NH4)2HPO4. Finally, 20 μl PEI solution (2 mg ml−1) (Wako) was added.

CaP/DNA/CaP/Protamine nanoparticles (control group) were prepared in an identical manner, with the final addition of 20 μl of protamine (10 mg ml^−1^) (Wako).

Single shell non-functionalized CaP/DNA nanoparticles (control group) were prepared by mixing 50 μl of an aqueous nCaP dispersion (prepared as above) with 20 μl of an aqueous solution of AcGFP1 or pUC57 plasmid in a sterile Eppendorf tube.

To prepare Lipofectamine^®^/DNA molecules (control group), 3 μl of Lipofectamine^®^ 2000 (1 mg ml^−1^) (Invitrogen; Life Technologies) was added to 75 μl DMEM (Wako) in a sterile Eppendorf tube. In another tube, 1.5 μl AcGFP1 plasmid solution was diluted in 75 μl DMEM. Both solutions were mixed together in a new Eppendorf tube and incubated at room temperature for 20 min.

PEI and protamine functionalized nCaP were fabricated as control groups like in our previous reports, where the loading of PEI or Protamine on nCaP surface increased the gene transfection efficiency [14,15]. Lipofectamine^®^ molecules were fabricated also as control group, as they are commonly used as transfection agents. All the prepared nanoparticles except the Lipofectamine^®^/DNA molecules were ultra-centrifuged at 40,000 rpm for 30 min (Himac CP80WX; Hitachi Ltd.) and reconstituted in distilled water prior to administration to the cells.

### Characterization techniques

Characterization of all R8-functionalized DNA-loaded nCaPs was performed by scanning electron microscopy (SEM–JEOL JSM-6390LA; JEOL Ltd.) after gold-palladium sputtering, and transmission electron microscopy (TEM–JEOL JEM-2100F; JEOL Ltd.). The CaP nanoparticle size determination, ζ potential, and the colloidal dispersions were measured via dynamic light scattering (DLS) using a Zetasizer nanoseries instrument (ELSZ-2, Otsuka, Japan). DLS refers to the speed at which the particles are diffusing due to Brownian motion is measured by recording the rate at which the intensity of the scattered light (laser) fluctuates. The particle size rate refers to scattering intensity distributions (ζ -average).

### Cell culture and transfection

For the transfection experiments, human transformed cervix epithelial cells (HeLa) (TaKaRa Bio Co.), primary osteosarcoma (Saos-2) cells (TaKaRa), hMSCs (Cellular Engineering Technologies), and hOBs derived from hipbone (PromoCell GmbH) were cultured in DMEM, supplemented with 10% foetal bovine serum (HyClone^®^, Thermo Scientific), and 1% Pen Strep (10,000 U ml^−1^ penicillin / 10,000 μg ml^−1^ streptomycin) (Gibco; Life Technologies) at 37°C in a humidified atmosphere with 5% CO_2_. Approximately 24 h prior to transfection, the cells were trypsinised and seeded in 24-well plates with a density of 2 x 10^4^ cells per well (number of wells per group n = 5).

Samples containing 50 μl nCaP dispersion were added to the cells in 450 μl new medium for a total volume of 500 μl per each well. This procedure was carried out for each kind of nCaP functionalized with R8, PEI, protamine. The same procedure was performed with the Lipofectamine^®^ molecules and single shell nanoparticles. After 7 h transfection, the medium was replaced with 500 μl new cell culture medium and the cells were incubated for an additional 72 h.

Transfection efficiency was determined after the period of incubation by transmission light microscopy and fluorescence microscopy (magnification ×100; BZ-9000; Keyence). Transfection efficiency was calculated based on the ratio of the fluorescing cells in which AcGFP1 was expressed, to the total number of examined cells. Dead cells, recognized by their round shape and floating in the medium, were not included in the computation.

### Cell viability test

The cell viability was analysed using the MTT assay (3-(4,5-dimethylthiazol-2-yl)-2,5-diphenylte-trazolium bromide) (Sigma-Aldrich Co.) 72 h after transfection. HeLa, Saos-2, hMSC, and hOB cells were seeded in 96-well plates at a density of 5 × 10^3^ cells per well approximately 24 h prior to transfection (number of wells per group n = 8). Transfection was conducted by adding 10 μl prepared nCaP to cells and 90 μl cell culture medium for a total volume of 100 μl. This procedure was carried out for each kind of nCaP functionalized with R8, PEI, or protamine. The same procedure was performed with Lipofectamine^®^ molecules and single shell nanoparticles. After 7 h of transfection, the medium was replaced with 100 μl new cell culture medium and the cells were incubated for an additional 72 h.

MTT was dissolved in PBS (4 mg ml^−1^) and then diluted to 1 mg ml^−1^ in cell culture medium. The cell culture medium of the transfected cells was replaced with 100 μl MTT solution and incubated for 1 h at 37°C under 5% CO_2_ in a humidified atmosphere. Then, the MTT solution was replaced with 100 μl DMSO. After 30 min, a 100 μl aliquot was taken for spectrophotometric analysis using a multi-scan microplate reader (SpectraMax^®^190; Molecular Devices) at λ = 570 nm. The absorption of the transfected cells was normalized to that of control (untransfected) cells, thereby indicating the relative level of cell viability.

### Determination of intracellular pathway

All cell lines were divided into groups and plated in 96-well plates with 5 x 10^3^ cells per well in 100 μL DMEM for 24 h prior to the uptake experiments (number of wells per group n = 8). Each group of cells was pre-incubated with different endocytosis inhibitors: sucrose as a clathrin-dependent endocytosis inhibitor [21] (0.4 M) (Wako) and LY294002 as a macropinocytosis inhibitor [22] (50 mM) (Cell Signaling Technology) for 30 min; and metil-β-cyclodextrin as a calveolae-dependent endocytosis inhibitor [23] (2 mM) (Tokyo Chemical Industry Co.) and amiloride as a macropinocytosis inhibitor [24] (5 mM) (Toronto Research Chemicals) for 10 min. After pre-treatment, 10 μl of the prepared nCaP was added to each group of cells along with 90 μl of D-medium for a total volume of 100 μl, followed by incubation for 4 h. This solution was then transferred to the ELISA assay plate, processed, and analysed by spectrophotometry using a multi-scan microplate reader at λ = 450 nm, according to manufacturer instruction. The procedure was carried out for each kind of nCaP functionalized with R8, PEI, or protamine and single shell nanoparticles in each cell line. A control group was established for each cell line for which no pre-treatment was administered. Any significant decrease of the BMP-2 concentration in a specific pre-treated group of cells compared to the non-pre-treated one, showed the intake mechanism of the nanoparticles into the cells.

### Intracellular pH measurement

The intracellular pH of the cells was measured using the intracellular fluorescent probe BCECF-AM (Dojindo Laboratories, Ltd.) according to the manufacturer’s protocol. Cultured cells were suspended in HEPES buffer solution (153 mM NaCl, 5 mM KCl, 5 mM glucose, and 20 mM HEPES) (pH 7.4) at 1 × 10^7^ cells ml^−1^. Membrane-permeable BCECF-AM (1 mM) was added to the cell suspension, which was then incubated at 37°C for 30 min. After the cells were washed three times with HEPES, the cells were resuspended at 1 × 10^6^ cells ml^−1^ in HEPES with various stimulants divided into two groups, the first including CaP/DNA/CaP/R8 at 10 mg ml^−1^ only and the second including amiloride and CaP/DNA/CaP/R8 at 10 mg ml^−1^, for 10 min at 37°C. The fluorescence intensity of BCECF-AM was monitored using a spectrophotometer (SpectraMax^®^M2e; Molecular Devices) with an excitation wavelength of 500 nm and a fluorescence wavelength of 530 nm. Intracellular pH was calibrated at different pH values between 6.6 and 8.2 using a calibration buffer (130 mM KCl, 10 mM NaCl, 1 mM MgSO_4_, and 10 mM Na-MOPS), with the addition of nigericin (Invivogen; Nacalai Tesque) at a final concentration of 10 μg ml^−1^.

### Statistical analysis

The results are summarized as percentage/mean ± standard deviation. Data were statistically analysed by Student t-test using IBM SPSS Statistics software (IBM Corp.). Statistically significant differences were defined as p < 0.05.

## Results and discussion

Recent studies have attempted to capitalize on the excellent biocompatibility and biodegradability properties of nCaPs compared to other inorganic nanoparticles and viral vectors25 by converting nCaPs into a multi-shell nanoparticle (CaP/DNA/CaP/DNA) to protect DNA from degradation by DNase and further enhance the transfection efficiency by the addition of various peptides [13,14,25]. Here, to address the remaining issues of peptide-related cytotoxicity and cell specificity, an out-layer of R8 was added to the multi-shell CaP nanoparticle and its effects on the transfection efficiency and cell viability of different cell lines were evaluated.

### Multi-shell octa-arginine functionalized DNA-loaded CaP nanoparticles

**Fig 1:**
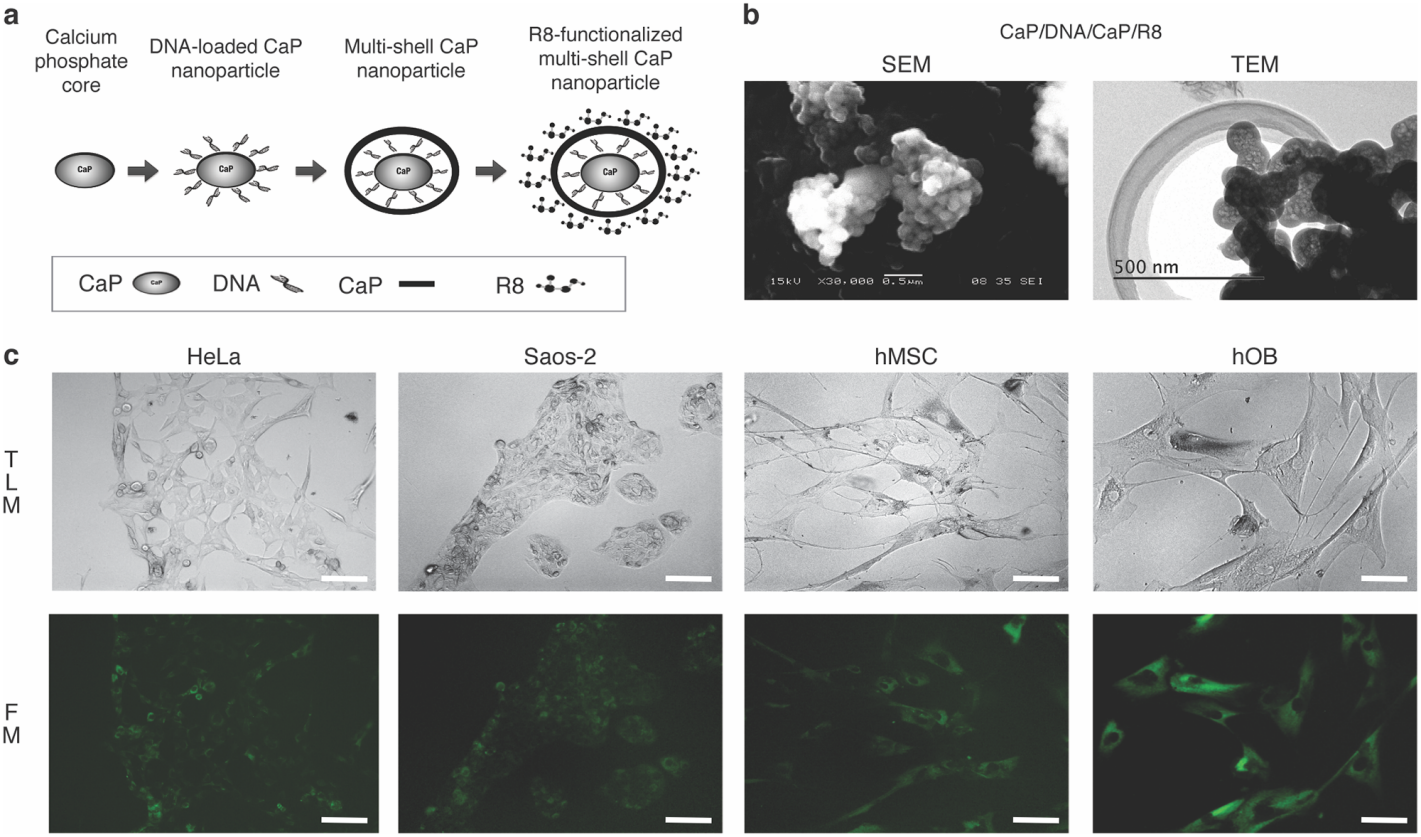
Gene transfection with octa-arginine functionalized DNA-loaded CaP nanoparticles. **a)** Schematic concept of fabrication of the octa-arginine-functionalized calcium phosphate nanoparticles. **b)** Scanning electron micrograph (SEM) and transmission electron micrograph (TEM) of CaP nanoparticles functionalized with octa-arginine (CaP/DNA/CaP/R8). **c)** Transmission light microscopy (TLM) and fluorescence microscopy (FM) of transfected cells. Representative images of HeLa, Saos-2, hMSC, and hOB cells transfected with the pAcGFP1 plasmid within CaP nanoparticles functionalized with octa-arginine at a concentration of 50 mg ml^−1^. Transfected cells appear green under fluorescence microscopy. Magnification: x20. Bar = 100 μm.

In this study, DNA-loaded nCaPs that were functionalized with different concentrations of R8 were fabricated (CaP/DNA/CaP/R8; Fig 1a). In addition, as the difference in the additional peptide on the outer-layer of the nCaP has been shown to influence the transfection efficiency and cell viability; i.e., the PEI-functionalized gene vector and the protamine functionalized gene vector, both found to exhibit high transfection efficiency [15,26], therefore PEI and protamine functionalized nCaP were fabricated as control groups. Other suitable control groups were Lipofectamine^®^ 2000, as a common cationic-lipid transfection agent, and single shell non-functionalized nCaP.

All functionalized nCaPs were characterized by dynamic light scattering (Table 1). The composition of all prepared nanoparticles was calculated in accordance with the method of Sokolova *et al*. [27]. The final concentrations are shown in Table 2 where we can see that only the outer-layer varied depending on the type of functionalizing peptide. The final concentration of CaP core and second shell of calcium phosphate vary depending on the volume of solution; The CaP core and second shell consists of 47wt.%, 27 wt.%, respectively. About the solubility, Sokolova *et al*. [27] has reported that the difference of intracellular calcium concentration between before and after the gene transfection using triple-shell calcium phosphate nanoparticles was very small with high cell viability and assumed an increase in the calcium concentration by 13 μmol L^-1^. In general, an increase of intracellular calcium concentration can lead to cell death. In this research, the cell viability was higher and corresponded with previous reports. This may be due to efficient cellular calcium extrusion mechanisms allowing to balance the still comparatively low nanoparticles mediating the calcium intake. Images taken by scanning electron microscopy and transmission electron microscopy (Fig 1b) showed a typical spherical morphology of the nanoparticles as previously reported [5,12]. The diameter of the particles from all samples was approximately 220–580 nm with a dispersion range of 0.234–0.866 nm. Sokolova *et al*. revealed that by measuring the particle ζ potential (a qualitative measure of the particle charge) while alternating the layer structure of the nanoparticles, the multi-shell structure of nCaP could be shown [12,13,27]. In this study, all functionalized nCaPs carried a positive charge, suggesting that R8, protamine, and PEI molecules, which have positive charge, were each present on an external layer of the nanoparticles, granting them a multi-shell structure. On the contrary, single shell non-functionalized nCaP carried a negative charge, which was granted by the plasmid layer over the CaP core. Additionally, for nCaPs functionalized with R8, the positive charge increased with the concentration of R8. Notably, the positively charged nanoparticle surface was found to assist with nCaP attachment to the negatively charged cell membrane [14,28,29].

**Table 1:** Characterization of functionalized calcium phosphate nanoparticles.

Nanoparticles	PDI	Average Size (nm)	ζ-Potential (mV)
CaP/DNA/CaP/R8 (0.1 mg ml^−1^)	0.234	309	+0.16
CaP/DNA/CaP/R8 (1 mg ml^−1^)	0.395	220	+1.53
CaP/DNA/CaP/R8 (5 mg ml^−1^)	0.270	315	+0.78
CaP/DNA/CaP/R8 (10 mg ml^−1^)	0.301	294	+13.60
CaP/DNA/CaP/R8 (50 mg ml^−1^)	0.866	506	+20.49
CaP/DNA/CaP/R8 (100 mg ml^−1^)	0.330	409	+20.90
CaP/DNA/CaP/PEI	0.295	580	+23.61
CaP/DNA/CaP/Prota	0.284	424	+13.57
CaP/DNA	0.327	255	-22.60

PDI: Polydespersity index from dynamic light scattering.

**Table 2:** Final concentrations of calcium phosphate (CaP), DNA- AcGFP1 or pUC57, octa-arginine, PEI, and protamine in nanoparticle dispersions.

Nanoparticles	CaP	DNA	Octa-arginine	PEI	Protamine	*(μg/well)*
CaP/DNA/CaP/R8 (0.1 mg ml^−1^)	22.78	3.45	0.11	0	0	
CaP/DNA/CaP/R8 (1 mg ml^−1^)	22.78	3.45	1.07	0	0	
CaP/DNA/CaP/R8 (5 mg ml^−1^)	22.78	3.45	5.36	0	0	
CaP/DNA/CaP/R8 (10 mg ml^−1^)	22.78	3.45	10.71	0	0	
CaP/DNA/CaP/R8 (50 mg ml^−1^)	22.78	3.45	53.55	0	0	
CaP/DNA/CaP/R8 (100 mg ml^−1^)	22.78	3.45	107.1	0	0	
CaP/DNA/CaP/PEI	22.78	3.45	0	2.14	0	
CaP/DNA/CaP/Prota	22.78	3.45	0	0	10.71	
CaP/DNA	12.19	3.45	0	0	0	

### Octa-arginine improved the transfection efficiency of the CaP nanoparticles with a high cell viability

The difference of R8 concentration on each multi-shell nCaP led to different gene transfection activities and cell viabilities among HeLa, Saos-2, hMSC, and hOB cells (Table 3) upon incubation together with the nanoparticles and as observed by transmission light microscopy and fluorescence microscopy (Fig 1c). In HeLa cells, the transfection efficiency of CaP/DNA/CaP/PEI was 54%, consistent with previous findings by Sokolova *et al*. (48%) [13]. In contrast, the transfection efficiency obtained using R8-functionalized nCaP was acceptable for the CaP/DNA/CaP/R8 nanoparticles at 0.1, 10, and 100 mg ml^−1^ concentrations, showing 17%, 19%, and 15%, respectively; however, these were significantly lower compared to that of nCaps functionalized with PEI and Lipofectamine^®^. Non-functionalized CaP/DNA nanoparticles showed the lowest transfection in the HeLa group with 4%.

**Table 3:** Transfection efficiency (TE) and cell viability (CV) in HeLa, Saos-2, hMSC, and hOB cells.

Nanoparticles	HeLa		Saos-2		hMSC		hOB	
	TE%	CV%	TE%	CV%	TE%	CV%	TE%	CV%
CaP/DNA/CaP/R8 (0.1 mg ml^−1^)	17 ± 6*	87 ± 7	11 ± 3*	99 ± 10	15 ± 5*	72 ± 9*	30 ± 6	77 ± 7*
CaP/DNA/CaP/R8 (1 mg ml^−1^)	6 ± 6*	88 ± 6	6 ± 2*	106 ± 5*	48 ± 4	76 ± 3*	38 ± 8	66 ± 4*
CaP/DNA/CaP/R8 (5 mg ml^−1^)	11 ± 5*	93 ± 6	3 ± 2*	111 ± 7*	48 ± 4	76 ± 10*	29 ± 9	66 ± 4*
CaP/DNA/CaP/R8 (10 mg ml^−1^)	19 ± 4*	81 ± 16	16 ± 3*	100 ± 11	31 ± 5*	74 ± 9*	40 ± 5	69 ± 8*
CaP/DNA/CaP/R8 (50 mg ml^−1^)	10 ± 4*	84 ± 8	26 ± 6	81 ± 5*	55 ± 4	88 ± 7*	38 ± 1	65 ± 6*
CaP/DNA/CaP/R8 (100 mg ml^−1^)	15 ± 4*	93 ± 8*	23 ± 5	100 ± 4	62 ± 5*	85 ± 5*	47 ± 6	83 ± 5*
CaP/DNA/CaP/PEI	54 ± 6	82 ± 8	31 ± 4	96 ± 6	47 ± 4	39 ± 5	36 ± 7	48 ± 5
CaP/DNA/CaP/Prota	13 ± 5*	86 ± 5	26 ± 3	103 ± 14	6 ± 2*	53 ± 7*	35 ± 3	47 ± 14
CaP/DNA	4 ± 3*	41 ± 6*	5 ± 1*	96 ± 15	6 ± 6*	82 ± 10*	3 ± 3*	58 ±6
Lipofectamine/DNA	41 ± 10	42 ± 11*	17 ± 3*	84 ± 15	77 ± 4*	19 ± 5*	29 ± 6	45 ± 5

*p<0.05 compared to CaP/DNA/CaP/PEI within each group

Notably, the cell viability of CaP/DNA/CaP/PEI was 82%, markedly higher than that obtained in previous report (23%) [15]. The cytotoxic effects of PEI are attributed to the adsorption of free PEI aggregates on the surface of the cell membrane and its rupture because of the high positive charge density of PEI [30,31]. This result suggests that the centrifugation step during the preparation of the nanoparticles might remove excess PEI molecules, thus reducing cell cytotoxicity. In comparison, the cell viability of CaP/DNA/CaP/R8 was similar or higher to that of CaP/DNA/CaP/PEI. The transfection efficiency and cell viability of CaP/DNA/CaP/Protamine were 13% and 86%, respectively, similar to those of the CaP/DNA/CaP/R8 groups. Conversely, the cell viability of Lipofectamine^®^ and CaP/DNA was 42% and 41% respectively, the lowest among the tested groups.

The transfection efficiency (26%) in Saos-2 cells obtained with CaP/DNA/CaP/R8 at 50 mg ml^−1^ was the highest among its group and showed no significant difference compared to that of CaP/DNA/CaP/PEI with 31%. The transfection efficiency of CaP/DNA/CaP/Protamine was also at 26%; these groups all exhibited high transfection compared to that of Lipofectamine^®^ and single shell nCAPs. Furthermore, cell viability was high in all groups of Saos-2 cells. In turn, for hMSCs, almost all groups demonstrated high transfection efficiency ranging between 31% and 62% with the exception of CaP/DNA/CaP/R8 at 0.1 mg ml^−1^. CaP/DNA/CaP/R8 at 100 mg ml^−1^ exhibited even higher transfection efficiency than CaP/DNA/CaP/PEI at 62%. Additionally, the cell viability of hMSCs treated with R8-functionalized nCaPs was 2-fold higher than that of the CaP/DNA/CaP/PEI group. For hOBs, no significant differences in transfection efficiency were observed between any CaP/DNA/CaP/R8 or CaP/DNA/CaP/PEI nanoparticle concentrations, whereas that for CaP/DNA/CaP/R8 at 100 mg ml^−1^ (47%) was higher than those nanoparticles functionalized with protamine and Lipofectamine^®^ molecules. Furthermore, all groups of R8-functionalized nCaPs resulted in higher cell viability than nanoparticles containing PEI, protamine, and Lipofectamine^®^ groups. In the case of single shell nCaPs, despite the cell viability was higher than that of PEI functionalized nanoparticles in both hMSC and hOB cells, the transfection efficiency of such nanoparticles was again the lowest among all groups with just a 6% and 3% respectively. This confirms the need of a multi-layer structure to improve their transfection efficiency.

Overall, there was a difference in the transfection efficiency and cell viability in these results that was related to the various cell lines and the functionalizing outer-layer of the nanoparticles. Previously, it has been reported that a gene transfer vector including PEI exhibited high transfection efficiencies on tumour cell lines, such as HepG2 and PC3 cells [32]; moreover, a gene transfer vector including R8 showed high transfection efficiency on both tumour cell lines including HeLa and A549 as well as non-tumour cells such as NIH3T3 [33]. In this study, the transfection efficiency and cell viability of PEI-functionalized nCaPs were higher in HeLa cells compared to hMSC and hOB cells. In contrast, the transfection efficiency and cell viability of R8-functionalized nCaPs were higher in hMSC and hOB cells compared to tumour cell lines regardless of R8 concentration. Although there was not a linear increase in the transfection efficiency of the nanoparticles with the increase of the concentration of R8, a substantial higher concentration of R8 correlated with higher transfection efficiency, as observed in CaP/DNA/CaP/R8 at 100 mg ml^−1^ particularly in hMSC, compared to that obtained with PEI. This was closely related to the ζ potential value of such nanoparticles, where nCaP functionalized with R8 100 mg ml^−1^ exposed a high positive charge of +20.90 mV, which may have increased the interaction with the negatively charge plasma membrane of the cells [14,28].

Furthermore, although the amino acid sequence of protamine is rich in arginine [34], the transfection efficiency of protamine-functionalized nCaP for hMSCs was lower than that of nCaPs functionalized with R8, whereas the efficiencies were similar in other cell lines. Futaki *et al*. reported that eight residues of arginine represented the most optimal sequence for successful transfection among the range of 4–16 arginines as determined using mouse macrophage RAW264.7 cells [17]. Thus, arginine sequence might exert considerable influence on gene transfection. Notably, nCaPs functionalized with R8 exhibited higher cell compatibility compared to that obtained with PEI or Lipofectamine^®^ in hMSC and hOB cells and did not demonstrate a concentration-dependent cytotoxic effect, resulting from the good biocompatibility of R8 [2,19,20]. Taken together, these results propose that the eight-arginine sequence improves transfection efficiency along with providing high cell compatibility of nCaPs in hMSC and hOB cells.

### The intracellular pathway varied widely among cell lines, the type of agent on the outer layer of nCaPs or R8 concentration

Transfection efficiency depends on many parameters such as temperature [35], pH value [36,37], concentration of calcium phosphate or DNA [38], cell line [15,33,39] and intracellular (endocytic) pathway [23,39]. In particular, the intracellular pathway identity and efficacy are expected to play important roles in defining the future applications of nanoparticles for transfection, gene silencing, imaging, and drug delivery [23,38]. Accordingly, the variant cell sensitivities for the respective functionalized nCaPs in this study might be caused by the difference of intracellular pathway. Therefore, the intracellular pathway of CaP/DNA/CaP/R8, /Protamine, and /PEI in each cell line was investigated by evaluating the concentration of released BMP-2 in cell culture medium after transfection with nanoparticles loaded with a BMP-2 expression construct. The intracellular pathways of non-viral vectors such as nCaP have been studied in the past and it is known that the nanoparticle uptake by the cells occurs through different endocytosis mechanisms, such as clathrin- and calveolae-dependent endocytosis and macropinocytosis [40].

We observed that the endocytic mechanisms utilized by the nanoparticles to enter the cells differed for each cell line and that even within the same cell line. In many groups, the pathway was not only one mechanism, but two. From all results, in each cell line, the relation between the pathway of nCaPs into each cell and the peptide, the concentration of the most out-layer, its size, and charge was not clear. This might be due to the fact that endocytosis is dependent on many different factors, including the type of cells, the nature of the carrier, particle size, pH, etc. Furthermore, the type of endocytic pathway utilized is dependent on the cell line [39], the nature of the gene carrier and the particle size [41]. All of these factors influenced the behaviour of the R8-functionalized nCaPs used in our study upon cell entry.

**Fig 2:**
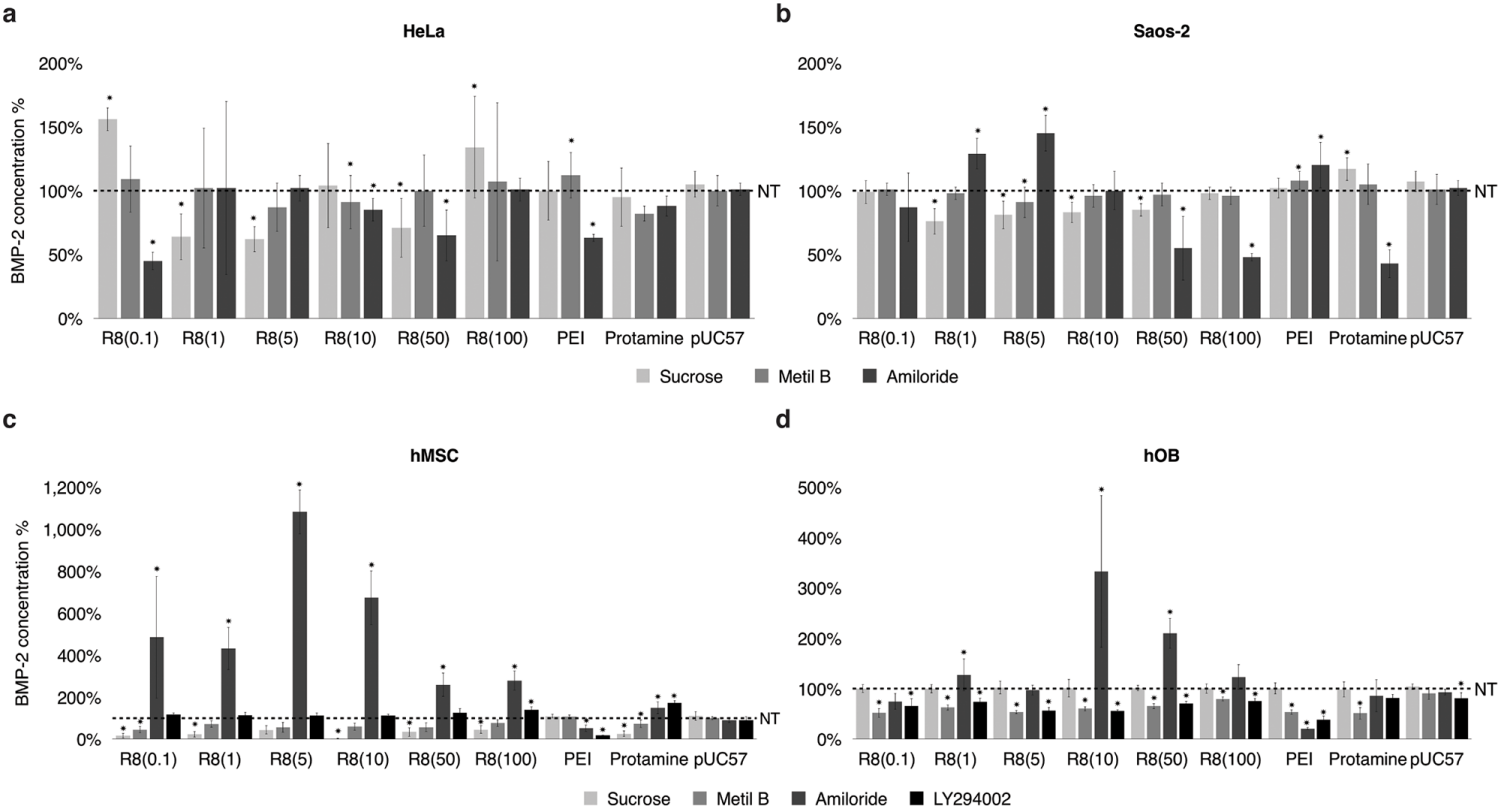
BMP-2 concentration percentages in the cell culture medium of cells pre-treated with endocytosis inhibitors. **A)** HeLa, **b)** Saos-2, **c)** hMSC, and **d)** hOB cells transfected with CaP nanoparticles loaded with pUC57 plasmid and functionalized with different concentrated solutions of R8 (0.1, 1, 5, 10, 50, 100 mg ml^−1^), PEI, and protamine. pUC57 group are single shell non-functionalized CaP nanoparticles. The bars represent the mean ± standard deviation. *p < 0.05 compared to transfection of non-pre-treated cells (NT).

For HeLa cells, the intracellular pathway utilized by CaP/DNA/CaP/R8 varied depending on the concentration of R8 (Fig 2a); i.e. 0.1, 10, and 50 mg ml^−1^ R8-functionalized nCaPs exhibited macropinocytosis—type uptake, whereas 1 and 5 mg ml^−1^ R8 showed clathrin-dependent endocytosis uptake, and 100 mg ml^−1^ R8 did not use any classical pathway. In comparison, CaP/DNA/CaP/PEI nanoparticles used macropinocytosis, which corresponded with the findings reported by Sokolova *et al*. wherein the same concentration of PEI had been added as the nCaP outer layer [38]. Finally, CaP/DNA/CaP/Protamine nanoparticles used both calveolae-dependent endocytosis and macropinocytosis.

Conversely, in Saos-2 cells, CaP/DNA/CaP/R8 alternated pathways with increasing concentration of R8 (Fig 2b). nCaPs including a low concentration of R8 utilized clathrin-dependent endocytosis, whereas CaP/DNA/CaP/R8 50 and 100 mg ml^−1^ nanoparticles changed to the macropinocytosis pathway. CaP/DNA/CaP/PEI nanoparticles did not use any classical pathway in these cells and CaP/DNA/CaP/Protamine nanoparticles incorporated the macropinocytosis pathway as well.

In hMSC cells, CaP/DNA/CaP/R8 nanoparticles utilized both clathrin- and calveolae-dependent endocytosis (Fig 2c). In contrast, the concentration of released BMP-2 in the hMSC group pre-treated with the endocytosis inhibitor amiloride was significantly elevated (*p<0.05), up to ten-fold higher in the cells transfected with CaP/DNA/CaP/R8 nanoparticles at 5 mg ml^−1^. For this cell line, we also pre-treated the cells with the LY294002 inhibitor, which inhibited macropinocytosis, and indicated that CaP/DNA/CaP/R8 nanoparticles did not use macropinocytosis as an intracellular pathway.

In hOBs, the concentration of released BMP-2 was reduced in all cells transfected with CaP/DNA/CaP/R8 nanoparticles and pre-treated with metil-β-cyclodextrin or LY294002, suggesting that the nanoparticles entered the cells by calveolae-dependent endocytosis as well as by macropinocytosis (Fig 2d). CaP/DNA/CaP/PEI nanoparticles also used both calveolae-dependent endocytosis and macropinocytosis and CaP/DNA/CaP/Protamine nanoparticles used calveolae-dependent endocytosis. Notably, the hOB cells pre-treated with amiloride also showed up to a four-fold increase in the concentration of released BMP-2 in the group transfected with CaP/DNA/CaP/R8 10 mg ml^−1^ nanoparticles. Furthermore, gene transfection with CaP/DNA/CaP/R8 into hOBs was inhibited by LY294002 but not by amiloride, although both act as macropinocytosis inhibitors. The uptake inhibition in hOB cells by LY294002 suggested that the CaP/DNA/CaP/R8 nanoparticles were taken up by macropinocytosis.

The arranged nCaP, having the size from smallest to largest are R8(1 mg ml^−1^), CaP/DNA, R8(10 mg ml^−1^), R8(0.1 mg ml^−1^), R8(5 mg ml^−1^), R8(100 mg ml^−1^), Protamine, R8(50 mg ml^−1^) and PEI in this study, On the other hand, the arranged nCaP having ζ-Potential from high positive to high negative are PEI, R8(100 mg ml^−1^), R8(50 mg ml^−1^), R8(10 mg ml^−1^), Protamine, R8(1 mg ml^−1^), R8(5 mg ml^−1^), R8(0.1 mg ml^−1^) and CaP/DNA. However, this sequence was not related with the pathway in all cell lines. The peptide, the concentration of the most out-layer, the size or ζ-potencial of nCaPs had no influence on the pathway of nCaPs, independently.

### Amiloride enhanced transfection in hMSC and hOB with a low influence in intracellular pH

Amiloride has been reported as an inhibitor of macropinocytosis [1,24] and Khalil *et al*. demonstrated that gene transfection using R8-modified liposomes were inhibited by amiloride in the NIH3T3 cell line [33]. In contrast, this study showed that the process of macropinocytosis was not inhibited in the pre-treated hMSC and hOB cells; instead, the concentration of released BMP-2 from the cells transfected with R8-functionalized nCaPs significantly increased (Fig 2c and 2d).

**Fig 3:**
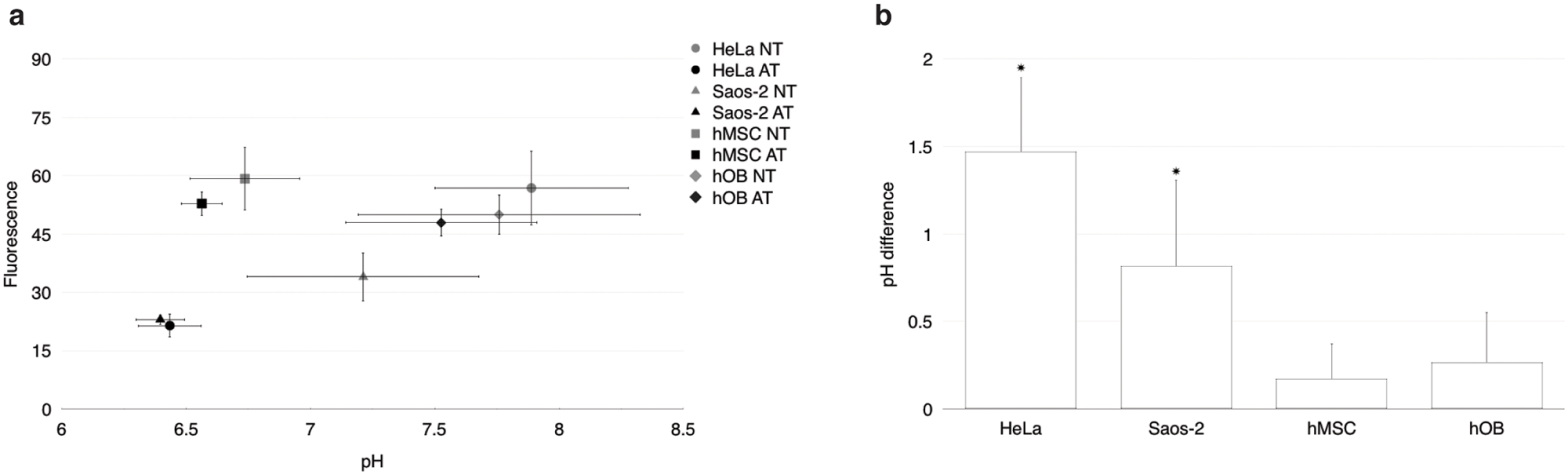
Influence of the endocytosis inhibitor amiloride on intracellular pathways. **a)** Intracellular pH of transfected cells with (AT) and without (NT) amiloride pre-treatment. **b)** Difference of intracellular pH in transfected cells between non-treated vs. amiloride pre-treated cells. The plots in **a** and the bars in **b** represent the mean ± standard deviation. *p < 0.05 compared to the intracellular pH of hMSCs.

LY294002 and amiloride utilize different mechanisms to inhibit macropinocytosis. LY294002 was reported to inhibit macropinocytosis by interfering with phosphatidylinositol 3-kinases (PI3k) [22], whereas amiloride was reported to inhibit pH-dependent macropinocytosis by lowering intracellular pH [24]. The cytosolic pH might affect the transfection efficiency of nCaPs functionalized with R8; therefore, we decided to measure the intracellular pH in all cell lines. Fig 3a shows the intracellular pH of transfected cells with and without amiloride treatment. All cells exhibited a decrease in intracellular pH upon treatment compared with untreated cells. In HeLa cells, the pH decreased from 7.8 to 6.4; in Saos-2, from 7.2 to 6.3; in hMSCs, from 6.7 to 6.6; and in hOBs, from 7.7 to 7.5. However, only the differences observed in HeLa and Saos-2 cell lines were significant (*p<0.05) (Fig 3b). Notably, Na^+^/H^+^ exchangers (NHE) are ubiquitously expressed in all cells and play a critical role in intracellular pH and cell volume homeostasis [42,43]; furthermore, amiloride has been shown to non-specifically inhibit all NHE isoforms [24,42,44]. Thus, the acidification that results when excess H^+^ production is uncompensated by the regulatory action of the NHE impairs macropinocytosis [23,24]. Conversely, no irregular behaviour was observed in the cells upon pre-treatment with LY294002, as this compound acts through a different mechanism: i.e. the inhibition of Pl3k, and loss of this critical kinase function affects various cellular processes including macropinocytosis [22].

These results suggested that the inhibition of NHE might be associated with the improvement of R8-functionalized nCaP transfection efficiency. However, the mechanism of such improvement upon amiloride pre-treatment of hMSCs and hOBs was not clear in this study. Despite the known inhibitory effect of amiloride on this process, our results show an irregular response across all transfected cells. This variability might be explained by the presence of calcium provided by the nanoparticles. Wakabayashi *et al*. found evidence suggesting that Ca^2+^ activates NHE by observing that the Ca^2+^ ionophore ionomycin rapidly stimulated NHE activity in the Chinese hamster lung fibroblast cell line PS 120 by increasing its pH sensitivity [45]. Murao *et al*. also reported that the NHE1 was regulated by the intracellular Ca^2+^ concentration and indicated that an increase of intracellular Ca^2+^ concentration induced alkalization of intracellular pH via NHE1 in AT-II cells [46]. In this study, the decrease amount of intracellular pH in hMSCs and hOB using R8 functionalized nCaPs after pre-treatment with amiloride was lower than that of tumour cell lines Hela and Saos-2. The intracellular alkalization in hMSCs and hOB after pre-treatment with amiloride might suggests an increase of intracellular calcium ions and the recovery of activities of NHE1. Meanwhile, several NHE genes have been identified in the human genome so far [47]. The different transfection efficiency among cell lines might be caused by the reaction of different NHE isoforms. Moreover, this might have influenced the enhancement of transfection efficiency under the presence of R8 by improving the permeability of the cell membrane or the nuclear membrane.

Alternatively, the intracellular pH of hMSCs and hOBs showed a value of 6.6 and 7.5, respectively, suggesting that the high transfection efficiency of R8-functionalized nCaPs observed upon pre-treatment with amiloride was not due to a specified adequate intracellular pH value. Furthermore, the small difference in intracellular pH (less than 1) within these cells following treatment, linked with the ability of R8 to stimulate macropinocytosis [21] likely combined to influence the intracellular uptake of the R8-functionalized nCaPs, increasing the concentration of BMP-2 in the pre-treated non-tumour cells compared to that of tumour cells. For example, Koivusalo *et al*. showed that modest changes in pH (a difference of pH 7.8 to 6.8) produced marked, highly significant decreases in macropinocytic efficiency in the human epidermoid carcinoma cell line A431, whereas in some cases the uptake of biomolecules was largely unaffected at a pH of 6.8 and much more acidic values had to be reached before a sizable inhibition of macropinocytosis was detected [24]. These results suggest that in the case of hMSCs and hOBs, the pH difference did not reach a degree of acidity sufficient to completely inhibit the uptake of the R8-functionalized nCaPs. Furthermore, the endocytic pathway of R8-functionalized nCaP (10 mg ml^−1^), which induced high transfection efficiency, was found to comprise clathrin-dependent endocytosis in hMSCs and calveolae-dependent endocytosis or macropinocytosis in hOBs, which suggested that the inhibition of a special endocytic pathway may have not contributed to the high transfection efficiency of R8-functionalized nCaPs upon pre-treatment with amiloride.

## Conclusion

In this study, DNA-loaded nCaPs functionalized with R8 exhibited high cell compatibility with a low level of cytotoxicity. Furthermore, the transfection efficiency of R8-functionalized nCaPs was higher or comparable to that of PEI or protamine in hMSCs and hOBs. The mechanism of intracellular delivery of the nanoparticles was influenced by the cell line and the concentration of R8. The outer-layer of the nCaPs influenced their specificity, which should be taken into consideration when preparing the nanoparticles according to the specified target cells. R8-functionalized nCaPs induced a markedly high transfection efficiency in hMSCs and hOBs pre-treated with amiloride; however, the mechanism remained unclear. Further investigations into the mechanism underlying the high transfection efficiency of R8-functionalized nCaPs upon amiloride pre-treatment in hMSCs and hOBs are currently underway. Together, these results suggest that R8 functionalized nCaPs represent a potential option as non-viral vectors for various future clinical applications requiring targeted gene delivery.
